# Factors associated with knowledge on obstetric danger signs among women who gave birth within 1 year in Bahir Dar city administration, North West, Ethiopia

**DOI:** 10.1186/s13104-019-4212-5

**Published:** 2019-03-27

**Authors:** Azezu Asres Nigussie, Amanu Aragaw Emiru, Yeshalem Mulugeta Demilew, Eleni Admassu Mersha

**Affiliations:** 1 Department of Midwifery, College of Medicine and Health Sciences, Bahir Dar University, Bahir Dar, Ethiopia; 2 Department of Reproductive Health, College of Medicine and Health Sciences, Bahir Dar University, Bahir Dar, Ethiopia; 3 Department of Nutrition, College of Medicine and Health Sciences, Bahir Dar University, Bahir Dar, Ethiopia

**Keywords:** Knowledge, Women, Obstetric danger signs

## Abstract

**Objective:**

This study aimed to assess factors associated with knowledge on obstetric danger signs among women who gave birth within 1 year in North West, Ethiopia.

**Results:**

Overall, 37.9% of the respondents were knowledgeable. Women mention three and more key danger signs during pregnancy and after delivery were 15% and 18.5% respectively. Decision making power of women [AOR = 1.59, 95% CI 1.10, 2.29], starting antenatal visit lately [AOR = 3.1, 95% CI 1.63, 6.33], housewife [AOR = 2.15, 95% CI 1.25, 3.68], merchant [AOR = 2.01, 95% CI 1.05, 3.88], and government employees [AOR = 2.75, 95% CI 1.38, 5.49] were among the predictors of knowledge on obstetric danger signs.

## Introduction

Obstetrics danger signs (ODSs) are unexpected symptoms that can lead to maternal health complications which occur during pregnancy, childbirth and after delivery [1]. Most could be prevented or managed if the woman had aware of it and seek medical care without delays [2–5].

Thus, Safe Motherhood initiative advocates the provision of advice during antenatal care (ANC) about ODSs and how to seek medical care for pregnant women and their families when raising these sudden obstetrical complications. This is viewed as central strategy to reduce delays in seeking skilled care [6].

Evidence suggests that ensuring the accessibility and use of obstetric services, and raising the awareness of women about ODS could save 310,000 newborn lives a year, and also, improves early detection of problems and reduces the delay in decision to seek obstetric care. Improving knowledge of women on ODSs enhances utilization of skilled care and decreases pregnancy rates in low-income countries [7, 8].

While a number of countries have made substantial progress in reducing maternal and child mortality, the high rate of maternal and neonatal mortalities related to obstetric causes. However, are still of great concern for many sub-Saharan countries including Ethiopia where the Maternal Mortality Ratio (MMR) is declining steadily [9, 10].

The World Health Organization (WHO) estimates that, currently 287,000 women a year die of preventable complications related to pregnancy and childbirth; the majority of these (99%) occur in developing countries and, out of those, 51% occur in the Sub-Saharan region [11].

Though, the 2015 target of MMR for Ethiopia was 218/100,000 live births, the 2005 and 2011 Ethiopian Demographic Health Surveys (EDHS) reported MMR were still high 673 and 676 respectively [12, 13].

Also the report of mid-term review of the Health Sector Development Program (HSDP-III) in Ethiopia related to maternal and neonatal health, at the community level, indicated limited progress in educating communities regarding danger signs during pregnancy and child birth [14].

Low coverage of maternal health services and high rate of neonatal and maternal mortalities related to obstetric causes are still of great concern in Ethiopia [13]. Lack of awareness of on obstetric complications might be the reason for failure of women to attend health care facilities, which initiated us to study level of knowledge and associated factors on obstetric danger signs.

## Main text

### Methods

#### Study design and settings

Community-based cross-sectional study was conducted at Bahir Dar City Administration from March 1–30, 2013. The city is located approximately 565 km Northwest of Addis Ababa, capital city of Ethiopia. It has 12 rural and 9 urban kebeles. The projected total population of the city was 251,309. Of these, 30,877 were female in reproductive age. More than 47 health institutions were available in the city administration in different level [15].

#### Study participants

All mothers who gave birth prior to the study were the source population. This study included all women who gave birth within 1 year during the data collection period. Mothers who had severe health problems during data collection period were excluded.

#### Sample size determination

The sample size was determined using single population proportions, 95% confidence level, 4% marginal error, 30.4% of prevalence from previous study [16], design effect 2, and 10% non-response rate, the final sample size was 715.

#### Sampling and sampling procedure

Multi-stage sampling technique was applied to identify study participants. First, all the Kebeles (the smallest administrative unit in Ethiopia) were stratified into urban and rural. Two urban and three rural Kebeles were selected by simple random sampling. Then, total sample size was allocated proportionally on each kebele. Finally, systematic sampling technique was employed to select the study subjects.

#### Data collection tool and procedure

Structured interviewer-administered data collection formats were developed from different literatures. Ten diploma Midwife interviewers and four BSc nurse supervisors were recruited and trained for 2 days. Face to face interview was done at the participant’s house. The questionnaire was piloted on 5% and necessary adjustments were made.

The knowledge of ODSs was assessed by ten items questions such as: during pregnancy (severe vaginal bleeding, swollen hands/face, and blurred vision), delivery (severe vaginal bleeding, prolonged labor, convulsions, and retained placenta), and postpartum period (foul-smelling vaginal discharge, and high grade fever) which has yes no response. Score one was given if the response is correct and zero was given for wrong response. The sum of responses above mean was considered as knowledgeable [17]. The supervisors and investigators have checked the filled questionnaires on the spot and correction was made on a daily basis.

#### Operational definitions

Knowledgeable on pregnancy ODS—ability of women to mention at least two of the three key ODSs during pregnancy.

Knowledgeable on child birth ODS—ability of women to mention at least two of the four key ODSs during child birth.

Knowledgeable on ODS after delivery—ability of women to mention at least two of the three key ODSs after delivery.

Overall knowledgeable: the ability of participants to mention above the mean of the selected ten key commonest ODSs.

#### Data management and data analysis

The data entering and cleaning was done through Epi Info version 3.5.3 and transported to SPSS version 16 for analysis. Both bivariate and multivariate logistic regression analyses were applied. A *P* value < 0.05 was considered statistically significant at 95% confidence level. Backward stepwise logistic regression analysis was employed to determine the putative association of independent variables with the outcome variable.

### Results

#### Socio-demographic characteristics of the study participants

Out of a total of 715 women planned to be included in the study, 701 women were enrolled in this study, yielding a response rate of 98%. The mean age of the study participants was 28.6 years with SD of ± 6.6 years; three-fourth of them, 529 (75.5%) were in the age group of 20–34 years.

Regarding to residence, 555 (79.2%) the respondents were urban dwellers. Most of the women, 565 (80.6%) were Orthodox Christians. Majority, 687 (98%) of them belonged to Amhara ethnic group. Two hundred fourteen (30.5%) respondents didn’t attend formal education. Housewives were 430 (61.3%), and 351 (50.1%) study participants got < 881 Ethiopian birr monthly. More than six in ten, 437 (62.3%) women lived within family consisting up to four members (Table 1).

**Table 1:** Distribution of the study participants by socio demographic characteristics in in Bahir Dar city, North West, Ethiopia, March, 2013 (n = 701)

Characteristics	Number	%
Age in years		
< 20	27	3.8
20–34	529	75.5
35+	145	20.7
Residence		
Urban	555	79.2
Rural	146	20.8
Marital status		
Married/in union	603	86.0
Single/separated	98	14.0
Religion		
Orthodox	565	80.6
Muslim	121	17.3
Protestant	12	1.7
Catholic	3	0.4
Educational level of the mother		
No formal education	214	30.5
Primary education	301	42.9
Secondary education	108	15.5
Higher education	78	11.1
Occupational status of the mother		
House wife	430	61.3
Merchant	98	14.0
G/employed	75	10.7
Others^a^	98	14.0
Ethnicity		
Amhara	687	98.0
Others^b^	14	2.0
Monthly income in birr		
75–450 birr	195	27.8
451–880 birr	156	22.3
881–2580 birr	176	25.1
2580–9950 birr	174	24.8
Family size		
1–4	437	62.3
5–6	218	31.1
≥ 7	46	6.6

^a^Indicate that: student, daily labour

^b^Indicate that: Tigrie, Age, Negde woyito

#### Knowledge on obstetric danger signs

Severe vaginal bleeding was the most frequently identified ODS during pregnancy (81.6%), delivery (82.2%) and after delivery (85.3%). On the other hand convulsion was the least mentioned ODS during pregnancy (10%), delivery (7.3%) and after delivery (8.8%). Despite the problem few women knew retained placenta (59.6%) and prolonged labor (51.6%) as danger sign related to delivery (Table 2).

**Table 2:** Knowledge on obstetric danger signs during pregnancy, delivery and after delivery in Bahir Dar City, North West, Ethiopia, March, 2013 (n = 701)

Obstetric danger signs	Knowledge on obstetric danger sign					
	Pregnancy		Delivery		After delivery	
	n	%	n	%	n	%
Vaginal bleeding	572	81.6	576	82.2	598	85.3
Severe headache	151	21.5	157	24.4	136	19.4
Blurring of vision	157	22.4			134	19.1
Loss of consciousness	239	34.1	229	32.7	229	32.7
Swelling of face/hands	171	24.4			200	28.5
Convulsion	70	10	51	7.3	62	8.8
High grade fever	132	18.8	111	15.8	130	18.5
Difficulty in breathing	214	30.5			193	27.5
Severe weakness	127	18.1			113	16.1
Severe abdominal pain	105	15			137	19.5
Increase/decrease of fetal movement	229	32.7				
Premature rupture membranes	146	20.8				
Retained placenta			418	59.6		
Prolonged labour			362	51.6		
Offensive vaginal discharge					199	28.4

#### Knowledge on the key commonest obstetric danger signs

The respondents were able to mention two key ODSs related to child birth, 418 (59.6%) and 199 (28.4%) after delivery, only 157 (22.4%) of them identified two key ODSs during pregnancy. The overall result indicated that, 266 (37.9%) of the respondents were knowledgeable regarding to the key commonest ODSs.

#### Factors affecting knowledge on obstetric danger signs

The multivariable logistic regression analysis revealed that Government employee [AOR = 2.75, 95% CI 1.38–5.49], merchants [AOR = 2.01, 95% CI 1.05–3.88], housewives [AOR = 2.15, 95% CI 1.25–3.68], women decision to seek medical care [AOR = 1.59, 95% CI 1.10–2.29] and timing in antenatal service utilization [AOR = 3.21, 95% CI 1.63–6.33] were statistically associated with knowledge on OSDs (Table 3).

**Table 3:** Bivariate and Multivariate analysis of predictors of knowledge on obstetric danger signs among mothers who gave within 1 year in Bahir Dar City, North west, Ethiopia, March, 2013 (n = 701)

Variables	Knowledge on danger sign			
	Yes (n = 226)	No (n = 435)	COR 95% CI	AOR 95% CI
Marital status				
Married/in union	227	376	1.65 (1.06, 2.55)	
Single/separated	39	59		
Occupation status of the mother				
House wife	168	261	1.84 (1.14, 2.98)	2.15 (1.25, 3.68)**
Merchant	39	59	1.89 (1.04, 3.44)	2.01 (1.05, 3.88)*
G/employed	33	41	2.30 (1.22, 4.34)	2.75 (1.38, 5.49)**
Others (student, daily laborer)	26	74	1	1
Family size				
1–4	157	276	0.70 (0.31, 1.57)	
5–6	91	131	0.38 (0.15, 0.94)	
≥ 7	18	28	1	
Final decision to seek care				
Respondent	129	164	1.57 (1.11, 2.21)	1.59 (1.10, 2.29)*
Other family	137	271	1	1
Number of ANC visits				
0	24	45	1	
1	18	26	1.04 (0.48, 2.24)	
2	41	59	1.16 (0.46, 2.91)	
3	46	83	1.19 (0.62, 2.28)	
4	137	222	0.41 (0.18, 0.93)	
Time of 1st ANC visits (n = 630)	(n = 206)	(n = 424)		
1st trimester	99	235	1	1
2nd trimester	77	154	1.19 (0.83, 1.70)	1.31 (0.77, 2.24)
3rd trimester	31	34	2.16 (1.26, 3.72)	3.21 (1.63, 6.33)***

*COR* crudes odds ratio, *AOR* adjusted odds ratio

* Significant at P < 0.02; ** Significant at P < 0.01; *** Significant at P ≤ 0.001

### Discussion

The overall result showed that 37.9% of the respondents were knowledgeable on the key commonest ODSs. This result is higher than study in Egypt (26%) [18]. The difference could be attributed to the time gap between the studies. Additional explanation could be the emphasis given by Ethiopian government in the last 5 years to improving maternal health services program.

In our study 22.4% of women were aware of at least two key ODSs during pregnancy. This finding is consistent with the study conducted in Pakistan (22%) [19]. However, the result of this study is lower than the studies done in Kenya (67%) [20], Tanzania (26%), and Uganda, 52% [21, 22]. The discrepancy might be related to operational definition given for knowledge on ODSs. In the previous studies a woman was considered knowledgeable if she answered at least one danger sign unlike our study in which at least two danger signs were required to consider knowledgeable.

The finding of this study indicated that 59.6% of mothers were aware of ODSs during childbirth. This is higher than the study done in Southern Ethiopia (41.3%) [16]. This difference could be due to the time gap of the studies and geographic difference; while this study was done in city administration whereas the previous study included the whole district in which the majorities were rural dwellers.

In this study 28.4% of women were aware on ODSs of postpartum period. This result is lower than the study result in Tanzania (40%) and Uganda (72%) [21, 22]. The difference could be attributed to the difference in geographical area, health care system and socio cultural factors.

Severe vaginal bleeding was the most frequently reported ODS during pregnancy (81.6%), labor (82.2%) and post-partum period (85.3%). This is consistent with studies done in Southern Ethiopia [16], Kyrgyzstan and Tajikistan and Uganda [17, 22]. This could be related to the visible nature of severe vaginal bleeding which can be easily recognized by women. Despite the fact that prolonged labour is the top most cause of mortality in Ethiopia [13], however, low proportions of women (51.6%) were aware of prolonged labour in relative to severe vaginal bleeding. This could be due to cultural and religious beliefs. As many participants were Christians, they could view labour pain as the way God intended, so that they might remain at home for longer period.

Housewives, merchants and government employee were more likely to be knowledgeable than other occupations. This result was in line with the studies from Egypt and Zambia [19, 23]. Women who were authorized to seek medical care by their own decision were more likely to be knowledgeable than women for whom decision was made by other family members [*P *= *0.013*]. This result was consistent with study finding in Uganda [22]. The correlation of decision power and knowledge could be due to high household asset owner ship of women in which they were not waiting for permission of the relatives and pocket of their husbands.

Those mothers who initiate first ANC on third trimester of pregnancy were more likely aware of ODSs than those women whose first ANC visit was on the first trimester of pregnancy [*P *= *0.001*]. The possible explanation of this might be related to parity. In this study most women who start first ANC on third trimester were multiparous. Therefore, ANC and education in their previous pregnancies could have an effect. In addition, women could have given more attention for health information they got when they were near term.

### Conclusion

Women had limited knowledge on ODSs during all three periods, more particularly during pregnancy. Low decision making power to seek care, early antenatal visit, being student and daily laborer, were among the predictors of lack of knowledge on ODSs. Therefore, there is critical need for provision of information on ODSs during the ANC period to facilitate the recognition on ODSs and access to skilled attendance services. We recommend that further studies need to be conducted to address all the three delays factors in a longitudinal approach.

## Limitation

Being a cross sectional study we can’t test the effect of knowledge on better maternal and neonatal health outcome. Recall basis may be also the limitation of the study.

## Abbreviations

ANC: Antenatal Care
AOR: Adjusted Odds Ratio
COR: Crudes Odds Ratio
EDHS: Ethiopian Demographic Health Surveys
HSDP: Health Sector Development Program
MMR: Maternal Mortality Ratio
ODSs: Obstetrics Danger Signs
WHO: World Health Organization
